# A qualitative study of community pharmacists’ opinions on the provision of osteoporosis disease state management services in Malaysia

**DOI:** 10.1186/s12913-016-1686-x

**Published:** 2016-08-30

**Authors:** Jah Nik, Pauline Siew Mei Lai, Chirk Jenn Ng, Lynne Emmerton

**Affiliations:** 1 Department of Primary Care Medicine, University Malaya Primary Care Research Group (UMPCRG), Faculty of Medicine, University of Malaya, Kuala Lumpur, 50603 Malaysia; 2 School of Pharmacy, Curtin University, GPO Box U1987, Perth, WA 6845 Australia

**Keywords:** Community pharmacist, Disease state management services, Osteoporosis, Bone disease, Qualitative, Malaysia

## Abstract

**Background:**

Osteoporosis has significant impact on healthcare costs and quality of life. Amongst the models for collaborative disease state management services published internationally, there is sparse evidence regarding the role of community pharmacists in the provision of osteoporosis care. Hence, the aim of our study was to explore community pharmacists’ opinions (including the barriers and facilitators) and scope of osteoporosis disease state management services by community pharmacists in Malaysia, informing a vision for developing these services.

**Methods:**

Semi-structured individual interviews and focus groups discussions were conducted with community pharmacists from October 2013 to July 2014. Three trained researchers interviewed the participants. Interviews were recorded and transcribed verbatim. Data were analyzed thematically using an interpretative description approach.

**Results:**

Nineteen community pharmacists with 1–23 years of experience were recruited (in depth interviews: *n* = 9; focus group discussions: *n* = 10). These participants reflected on their experience with osteoporosis-related enquiries, which included medication counseling, bone density screening and referral of at-risk patients. Key barriers were the lack of numerous factors: public awareness of osteoporosis, accurate osteoporosis screening tools for community pharmacists, pharmacists’ knowledge on osteoporosis disease and medications, time to counsel patients about bone health, collaboration between pharmacists and doctors, and support from the government and professional body. The pharmacists wanted more continuing education on osteoporosis, osteoporosis awareness campaigns, a simple, unbiased osteoporosis education material, and inter-professional collaboration practices with doctors, and pharmacists’ reimbursement for osteoporosis care.

**Conclusions:**

The involvement of community pharmacists in the provision of osteoporosis disease state management was minimal. Only ad-hoc counseling on osteoporosis prevention was performed by community pharmacists. Development and trial of collaborative osteoporosis disease state management services in community pharmacy could be facilitated by training, support and remuneration.

## Background

Osteoporosis is an increasingly important health problem with significant impact on morbidity, mortality, quality of life and cost [1]. By the year 2050, almost 50 % of hip fractures worldwide will occur in Asia, due to the density of the elderly population in this continent [2]. In Malaysia, 7.1 % of the population will be aged 65 years or over by 2021 [3].

Despite an advancement in the diagnosis of osteoporosis, publication of clinical practice guidelines, development of screening and fracture risk assessment tools, and interventions to reduce the risk of fractures, only a minority of men and women with a high fracture risk receive treatment [4]. This may be due to healthcare professionals’ inertia in initiating treatment for osteoporosis, or their lack of knowledge [5], or a lack of awareness among the public of osteoporosis [6, 7]. Undermanaged osteoporosis incurs significant costs to primary and secondary care [8, 9]. As such, early detection and treatment of patients at risk are critical. Preventative measures at the community level are also urgently needed.

Pharmacists can play an important role in the provision of osteoporosis disease state management services [10]. This includes identifying and managing the risk factors for osteoporosis, providing counseling on nutrition, exercise and lifestyle relevant to bone health, identifying the role of calcium and vitamin D as preventative and concomitant therapy for those receiving osteoporosis treatment, discussing the various treatments available with advantages and disadvantages, and offering counseling to ensure optimum administration [10]. In addition, community pharmacists can offer bone density assessments with heel ultrasound [11, 12], or fracture risk prediction such as FRAX® [13]. This requires a multidisciplinary/inter-professional collaborative approach. Compared to other healthcare professionals, community pharmacists are generally in a better position to provide continuity of care due to their accessibility to patients [14–16].

A review of literature on osteoporosis disease state management found that several studies involving community pharmacists have been conducted in the United States [11, 12, 15, 17], Canada [13, 18], The Netherlands [19, 20], Australia [21] and Thailand [22]. Most published studies in the United States reported on the implementation and outcomes of osteoporosis management services in community pharmacies [11, 17]. However, details regarding the development of these services were sparse. The reported services comprised osteoporosis screening, counseling and referral to physicians [12, 15, 17], as well as risk-factor assessment [11]. These studies reported positive outcomes relating to osteoporosis awareness, prevention and patients’ medication adherence and referral of high-risk patients [11, 17]. However the reported outcomes did not encompass pharmacists’ confidence, acceptability and integration into work flow in delivering the osteoporosis services, but focused more on patients’ satisfaction. Research into the involvement of community pharmacists is critical to the conceptualization and acceptability of novel services.

There are specific challenges in developing disease state management services in a number of Asian countries. In Malaysia, patients can obtain their medicines directly from a doctor’s clinic, instead of having their prescriptions dispensed in a community pharmacy [23]. As a result, community pharmacists in Malaysia are not fully utilized for professional services [24]. In developed countries such as Australia and the United Kingdom, where medications are solely dispensed by pharmacists, community pharmacists have a greater impact on the community, and have more opportunity to provide disease state management services [25]. Given the predicted prevalence of osteoporosis in Asia, the deficiencies in studies relating to osteoporosis management services, and the challenges with establishing such services, our study aimed to address these gaps. The aim of our study was to explore the opinions and scopes of practice of community pharmacists in Malaysia in osteoporosis disease state management, and factors that influence their practices.

## Methods

### Design

In view of the exploratory nature of the research question, qualitative methodology was applied to enable collection of in-depth information to understand and interpret the personal experiences of community pharmacists in their interactions with clients, particularly osteoporosis patients, in their daily practice. These experiences were drawn on to identify the community pharmacists’ opinions (including their barriers and facilitators), and scope of the osteoporosis disease state management services. We used an interpretative descriptive qualitative approach, and an inductive analytical approach to “seek understandings of clinical phenomena that illuminate their characteristics, patterns and structure” [26]. This method of study was chosen to describe the phenomena from the perspective of respondents [27]. Our study was reported according the the consolidated criteria for reporting qualitative studies (Appendix 1).

### Setting and participants

This study was conducted in Malaysia, a multi-race country with a dual-sector health-care system comprising public government-subsidized health care and private health care, where most medications are dispensed from government hospital pharmacies, private health care clinics or hospitals, rather than from community pharmacies [28]. Most independent community pharmacies in Malaysia are privately owned by pharmacists, while chain pharmacies are owned by corporations.

We included full-time community pharmacists with a minimum experience of 1 year as a community pharmacist, and who were able to converse in either English or Malay. Inclusion of pharmacists with or without specific experience in managing osteoporosis enabled exploration of their perceived barriers to introducing this type of service. Locum pharmacists (defined as working fewer than 40 h per week) were excluded. Purposive sampling was used to achieve maximal variation based on two factors: years of community experience, and type of practice (as an independent pharmacist, or those employed in chain pharmacies). If self-employed, pharmacists in independent pharmacies would have more liberty to determine the way they practiced, as opposed to pharmacists employed in chain pharmacies.

An invitation to participate in this study was posted online via Facebook, LinkedIn and Eventbrite. The advertisement explained the objective and nature of the study. Pharmacists who expressed interest to participate were contacted by email, and their telephone number were obtained. The participant information sheet detailing the inclusion criteria for participants and researchers’ contact information was emailed to the respondents. Where feasible, pharmacists were allocated to one of two focus groups, scheduled in the morning over a period of 3 weeks at a convenient venue. Focus group discussions were conducted whenever possible to generate discussion among the participants through group dynamics [29]. Those unable to participate in one of the focus groups were offered an at-work one-on-one interview. The researcher then arranged the interview at a mutually-convenient date and time. At the end of the interviews or focus group discussions, participants were asked for any contacts they felt might be suitable, and if these suggested pharmacists were considered to meet the criteria for purposive sampling, they were contacted.

### Data collection

An interview topic guide (Table 1) was developed based on literature review, conceptual framework (described below) and expert opinion. A baseline demographic form was used to collect data on participants’ age, gender, education background and practice experience. All focus group discussions were facilitated by PSML or CJN, with JN in attendance as a note taker. In depth interviews were performed by PSML, CJN or JN. Discussions and interviews were audio-recorded, supplemented by note-taking for focus groups. Each in-depth interview lasted 30–60 min, whereas each focus group discussion lasted approximately 60 min. No repeat interviews were carried out.

**Table 1:** Interview topic guide

1.	What sort of pharmaceutical services do you provide?
2.	Do you think that the provision of pharmaceutical care would generate more income for you?
3.	What do you understand by the term “osteoporosis”?
4.	What would an osteoporosis management service in a pharmacy comprise?
5.	In your opinion, how aware do you think the general public is about osteoporosis?
6.	When did you last refer someone with osteoporosis to a doctor?
7.	Do you face any difficulty when dealing with people with osteoporosis?
8.	What would encourage you to provide osteoporosis disease state management services?
9.	If you were to start providing osteoporosis disease state management services, what form of support would you require?

### Theoretical framework

The integrated behavior model (IBM) was used as a conceptual framework to develop the topic guide for this study and assisted in the interpretation of data. The IBM proposes that people act on their intentions when they have the necessary skills and when environmental factors do not impede performance. As such, behavior can be influenced through changes in skills, environmental factors and behavioral intention [30]. Based on the IBM determinants, community pharmacists’ opinions toward the provision of osteoporosis disease state management services can be explored by interviewing pharmacists about their skills and knowledge relating to osteoporosis, their opinions about the importance of these practices and their environmental constraints. When people have formed appropriate intentions but are not acting on them, interventions can be developed to address skills or environmental barriers such as lack of public demand and lack of government and society support [31].

### Data analysis

Focus group discussions and interviews were transcribed verbatim for analysis. The transcripts were not returned to participants for comment or correction. Grammatical imperfections were retained to reflect the participants’ voices. A thematic analysis approach was used to analyze the data within the broad categories of ‘barriers’ and ‘facilitators’. Other relevant data relating to experiences with the provision of services were presented descriptively [27]. For the thematic analysis, four researchers initially worked in pairs (JN/CJN and PSML/LE) and coded two interviews line-by-line to develop an initial list of nodes. Using NVivo version 10 (QSR International Pty Ltd), this framework was then used to code the next transcript. Coding discrepancies were resolved by discussion between representatives of the pairs until consensus was reached. The lists of nodes were used as the final coding framework for the remaining transcripts. New nodes emerging during coding were added to the list upon consultation with the research team, and previously-coded transcripts were checked in terms of retrospective fit of these nodes. The lists of nodes were regrouped into larger categories as themes emerged from the data. This systematic approach to the analysis established an audit trail from the transcripts of raw data through to the final interpretation. Analysis was undertaken concurrently with data collection to check for data saturation [32]. Data collection ceased when thematic data saturation was reached. Data saturation is occurred when no new themes were perceived by the interviewers to emerge between interviews 16 and 19, and data collection was ceased following the 19th interview. Data analysis confirmed coherence of the emergent themes.

### Rigor and trustworthiness

Rigor during data collection was enhanced by recruitment of participants with maximal variation within our inclusion criteria, standardization of the interview questions (still allowing for adaption to the conversational flow), and use of trained interviewers. During analysis, interpretations were discussed between members of the research team. Involvement of multiple researchers has been recommended when conducting qualitative research, particularly to assist with data interpretation; multiple researchers may supplement and contest each other’s statements, which may enrich and qualify the analysis [33, 34].

## Results

Nineteen community pharmacists (28–54 years of age), with 1–23 years of experience as community pharmacists were recruited. Nine pharmacists underwent in depth interviews, whilst 10 pharmacists participated in focus group discussions. The majority were female (*n* = 12). All participants had a Bachelor degree in Pharmacy. Most of the pharmacists recruited (*n* = 13) were from independent pharmacies. Findings are presented in three sections: description of osteoporosis disease state management services components followed by the barriers and facilitators to this service. The quantification of nodes for each themes was presented in Appendix 2 to present the relative emphasis given by community pharmacists.

### Provision of osteoporosis disease state management services by community pharmacists

In this study, most community pharmacists did not proactively provide osteoporosis disease state management services. However, 13 pharmacists (10 from independent, 3 from chain pharmacies) reported receiving and responding to requests for information from patients about the risk factors for osteoporosis and preventative measures, and providing advice on weight-bearing exercise. Recognized risk factors were smoking, those who drink excessive amounts of alcohol and caffeine, those who are thin and small built, and use of medicines such as steroids or thyroid medication.

Most pharmacists felt it was important to counsel patients to improve adherence to medications and minimize side effects.

“.. *all the medications have side effects such as don*’*t lie down to minimize the risk of side effects*, *don*’*t drink coffee after you take Fosamax*® *and must be compliant*, *what happen if miss dose*, *you have to take it immediately*, *if too close*, *then skip the previous one*, *don*’*t double the dose*.” − 31-year-old female

### Screening using heel ultrasound

Of these, five independent community pharmacists reported having hosted an ultrasound machine in their pharmacies. Twelve pharmacists (10 from independent pharmacies and 2 from chain pharmacies) reported divergent views about the potential use of heel ultrasound to screen for osteoporosis in their practice. While some felt ultrasound screening would promote public awareness about osteoporosis and provide an opportunity for clients to ask pharmacists about osteoporosis, others were doubtful about the accuracy of the machine [in comparison to dual-energy x-ray absorptiometry (DXA)] and skeptical about the motive of the vendor. The participants were aware that it would not be cost-effective to purchase the ultrasound machine, and that screening was ultimately linked to sales of osteoporosis-related products such as calcium supplements.

“… *Bone scan on* (*the*) *ankle might not be that accurate*….. (*Screening for osteoporosis using heel ultrasound*) *is also a trigger point for them* (*clients*) *to discuss*… *what osteoporosis* (*is all about*)…”-54-year-old female

“…. *we can actually utilize the machine*, *but double edge is when suppliers come in they have something in their mind*. (*If*) *I* (*use*) *this machine*, *at the end of the day I would like to have some sales*…” – 30-year-old male

### Screening using osteoporosis questionnaires

One pharmacist from an independent pharmacy suggested that screening for osteoporosis can be provided in the community pharmacy. This can be performed by calculating the client’s osteoporosis risk score based on their age and weight.

“…. *I will just tell* (*customers*) *what are the causes*… *even actually osteoporosis*, *we can use and calculate based on your age and weight*, *osteoporosis risk score*, *you*’*ll tell them which category fall on*.”—30-year-old male

Despite the divergent views regarding the use of heel ultrasound screening, the pharmacists agreed that accessibility of community pharmacies was key to screening and educating the public about osteoporosis.

### Barriers to providing osteoporosis disease state management services

Eight themes emerged as barriers to the provision of osteoporosis disease state management services by community pharmacists: (1) lack of public awareness, (2) osteoporosis is a hidden disease, (3) high cost of osteoporosis medications, (4) lack of accurate and validated screening tools for osteoporosis to be used in community pharmacies, (5) lack of pharmacists’ knowledge (6) lack of time to counsel about bone health, (7) lack of collaboration between pharmacists and doctors due to the lack of dispensing separation, and (8) lack of continuity of care.

### Lack of public awareness

Currently, the main barrier faced by community pharmacists was the lack of demand for osteoporosis prevention from customers due to their lack of awareness regarding osteoporosis.

“*So far*, *we don*’*t really have* (*any*) *customer that comes to the pharmacy and say* “*I want to prevent osteoporosis*,” *but they come and say* “*I want to do blood sugar screening*” *or the other like cholesterol*, *but particularly for osteoporosis*, *nobody comes*….”—46-year-old female

The pharmacists perceived that the public’s lack of awareness regarding osteoporosis may be due to a lack of public campaigns regarding the severity of the osteoporosis, and its impact on health.

“… *One of the reason for me*, *osteoporosis is not something that I target is because there is lack of public campaign*… *If there is a public campaign*, *and then concurrent together* (*with*) *pharmacies then we will be able to get the numbers that we hope for*…” - 51-year-old female

### Osteoporosis is a ‘hidden’ disease

The pharmacists also perceived that the public was not aware of osteoporosis as it is a silent disease, unlike osteoarthritis, which is associated with pain.

“*I think because osteoporosis is actually a hidden kind of health condition*, *because most people don*’*t know they have osteopenia or osteoporosis until they do the scan or if they have a fracture. Not like other conditions sometimes you know they may have symptoms*”—39-year-old female

### High cost of osteoporosis medication

Pharmacists also perceived that patients do not prioritize osteoporosis as an important health condition. They perceived that patients would rather spend their money on medications for other health conditions such as osteoarthritis.

“… *sometimes it might be financial reason as well*, *and you know they may wanted to use the money for other most significant kind of condition than this*, *because they don*’*t feel the pain*, *maybe they will ignore until latest stage when it became serious*.”—39-year-old female

Osteoporosis medications can only be dispensed on a doctor’s prescription. However, due to the lack of dispensing separation in Malaysia, doctors are able to dispense osteoporosis medication directly to patients. As a result, community pharmacists seldom receive prescriptions to dispense osteoporosis medications from doctors. Consequently, five pharmacists reported they did not stock osteoporosis medications in their pharmacies.

“… (*Medications*) *are quite expensive and there*’*s no demand. I didn*’*t keep it* (*osteoporosis medications*) … *unless the customer*’*s request and she can provide prescription*, *then I*’*ll keep*.”—29-year-old male

### Lack of accurate and validated screening tools for osteoporosis in community pharmacy

Twelve pharmacists (10 from independent pharmacies and 2 from chain pharmacies) doubted the accuracy of available screening tools. They felt heel ultrasound was less accurate compared to a DXA scan, and preferred their at-risk clients to be diagnosed and monitored via DXA.

“…*of course we know* (*ultrasound is*) *not 100* % *accurate* … *for some customers who are more health conscious they also know. So*, *I will advise them to go to* (*the*) *hospital*…”—43-year-old male

On the other hand, these pharmacists felt ultrasound screening was important before discussing bone health. This would require full-time access to a heel ultrasound machine.

“… *When we want to discuss about bone health*, *sometimes they* (*customers*) *want to see some proofs. So*, *we must do some sort of test* … *the problem is we don*’*t have that machine*…”—42-year-old female

Pharmacists who had hosted ultrasound screening tended to rely on vendors of ultrasound machines to operate the machine, due to the high cost of that service.

“…*normally we just depended on the supplier to provide us with the screening test*, *unless we buy the machines for ourselves*, *then solve the problem*…”- 42-year-old female

### Lack of pharmacists’ knowledge

Some pharmacists were not confident to talk about osteoporosis with their customers because they did not know much about osteoporosis and its management.

“*I*’*m not that confident because I do not have a big picture of what is the treatment*, *what is the medicine*, *all the apparatus they are using*, *that one is too much doctor thing for me. So*, *the management part*, *I am not that familiar*.” - 37-year-old female

### Lack of time to counsel about bone health

Time was a perceived limitation to the provision of osteoporosis disease state management services; as such, screening and counseling about bone health was not a priority when the pharmacists were busy and clients presented with many health problems.

“…*then for us to attend the customers*, *we have to allocate special time to educate*, *to counsel the customers*, *it*’*s not touch and go thing. It*’*s like counseling anything about health. Time is the factor that I said*…”—42-year-old male

Additionally, this participant perceived that the provision of osteoporosis disease state management services was time consuming.

“…*osteoporosis counseling involves many things such as diet*, *exercise*, *exposure to sunlight*, *muscle mass*, *body weight and so on. We* (*will need*) *to talk about* (*this*) *for half an hour* …”—43-year-old male

### Lack of collaboration between pharmacists and doctors

The Malaysian healthcare system was not conducive in supporting pharmacists to work with doctors in providing patient care. Since both can dispense medications, there was a conflict of business interest between them.

“… *That*’*s the problem with our healthcare system* … *I think because both of us*, *doctors and pharmacists*, *can dispense*, *both doctors and pharmacists are doing business* … *conflict of interest* … *very seldom community pharmacists get prescriptions* (*from doctors*).” - 42-year-old female

This indirectly affected the provision of osteoporosis disease state management services by the pharmacists, who require doctors’ support in the diagnosis and treatment of osteoporosis as well as access to patients’ medical records.

“… (*osteoporosis*) *is quite familiar to me*, *but the problem is about the diagnostic part*, *yet to be confirmed by doctor*… *we might have some difficulties in order to know what stages they are*, *whether they have osteoporosis or not. To me*, *unless the clients are actually eager to know*, *we can share more*; *otherwise osteoporosis might be the part that everybody missed*.”—26-year-old male

Pharmacists, in general, felt it was difficult for them to initiate a conversation about osteoporosis when patients were not specifically referred to them for counseling.

“… *whenever customers come in complaining bone pain*, *knee pain*, *that is the time we start to get actively involve*, *usually as a pharmacist*; (*to initiate a*) *management plan or counseling*, *we depend on doctors*’ *diagnosis*…”—33-year-old male

### Lack of continuity of care

According to one pharmacist, customers tended to ‘shop around’ for cheaper medicines. The resulting lack of continuity of care posed a challenge to pharmacists in providing effective osteoporosis care for the patients.

“… *I notice that we have regular customers*, *but sometimes they shop around. They will go asking for prices at different pharmacy*, *and then they will buy this thing from this pharmacy because is about RM2 cheaper. So*, *they don*’*t actually stay at one pharmacy usually*.”—39-year-old female

### Facilitators in the provision of osteoporosis disease state management services

Six themes relating to facilitators were identified from the data. These themes were further subdivided into current facilitators and potential facilitators. Current facilitators included continuing education support, support from healthcare industry and availability of suitable materials. Potential facilitators included creation of public health campaigns, inter-professional collaboration and the provision of reimbursement mechanisms.

### Continuing education support on osteoporosis

Most pharmacists mentioned a need for support from professional societies, such as the Malaysian Pharmaceutical Society, via education talks and seminars to improve pharmacists’ knowledge on osteoporosis.

“…*We need to keep on educating ourselves*, *because there will be new knowledge on osteoporosis*, *new supplements*, *new results for taking calcium*, *new awareness. This support is very important for us*, *as a pharmacist*.”—42-year-old male

### Support from healthcare industries

Pharmaceutical industries can support pharmacy services by providing heel ultrasound machines to screen for osteoporosis and the latest information update on osteoporosis.

“…*I think screening provided by certain* (*pharmaceutical*) *company will encourage people to be more aware of this problem in my area. I believe this will trigger the awareness of osteoporosis*.”—47-year-old female

### A simple and unbiased educational material regarding osteoporosis for patients

Almost all pharmacists felt a simple and unbiased customers’ education material regarding osteoporosis would facilitate them to provide osteoporosis disease state management services. The education materials could either be a booklet or pamphlet produced by an independent body, and should contain unbiased information on osteoporosis without product promotion.

“… *if you have the booklet or anything about osteoporosis from independent sources*, *we can just give it to the customer when we are busy so they can read it*… *if it is from the* (*medical*) *company*, *definitely it will focus on their products*.”—33-year-old female

### Public health campaigns on osteoporosis

Although the involvement of community pharmacists in the provision of bone health was minimal, pharmacists considered that they could play a more significant role in health promotion and disease prevention, especially in osteoporosis. The pharmacists felt that awareness about osteoporosis should be promoted by the government through media and health education activities so the public would visit their pharmacists for advice about bone health and osteoporosis.

“…*the activities*, *we can go to school*, *where you can reach the young generation or offices*… *where the government can help us to penetrate the mass public*.” - 42-year-old male

### Inter-professional collaboration practices

Pharmacists felt strengthening inter-professional ties would improve the provision of osteoporosis disease state management services by community pharmacists. Ideally, the pharmacists would recommend high-risk patients to consult doctors for assessment, and the doctors would refer the patients to the pharmacists for osteoporosis care.

“…*basically we can*’*t work with one hand* … *collaboration with* (*the clinic or private doctors*) *will be quite a good one in the sense that you refer* (*patients*) *for DXA scan and it*’*s a win*-*win situation and* (*the doctors*) *get pay for their DXA and subsequently whatever they have prescribed or not*, (*patients*) *can always come back to the pharmacist*.”—30-year-old male

### Government reimbursement for pharmacists’ services

Most pharmacists felt that if they were paid by the government for their time spent with their customers, they would be more motivated to provide osteoporosis disease state management services.

“*Well*, *in my opinion* … *it is a good start* … *if we are reimbursed for counseling* … *that will motivate us very much*.”—42-year-old male

## Discussion

Findings from this limited sample suggest Malaysian community pharmacists’ involvement in the provision of osteoporosis disease state management services is minimal. Our cohort appeared to be deeply rooted in the traditional role of medication dispensing and counseling, although they recognized the potential to expand the boundaries of their work [35]. In this study, only ad-hoc counseling, such as advice on osteoporosis prevention, risk factors and lifestyle modification, was practiced. This was due to a lack of referral from doctors to pharmacists. Furthermore, the public has a low level of awareness of osteoporosis, as it is a silent disease until fractures occur [36]. One of the proposed public health messages, and indeed a cornerstone of a pharmacist-initiated service in this area, is the ‘latency’ of osteoporosis and the need for prevention and early detection. These messages would be used to introduce service components such as FRAX for screening. FRAX estimates fracture risk within 10 years. It is a validated tool for pharmacists’ risk assessment for osteoporosis, and thus is useful to educate patients about this condition. Community pharmacists also can play a more significant clinical role in identifying drug-induced osteoporosis, such as in corticosteroid users [37, 38]. Our participants also supported the role of community pharmacy in the management of diagnosed osteoporosis through counseling to optimize adherence to prescribed osteoporosis medicines and lifestyle changes.

Review of the literature indicates the prevalence of osteoporosis in Asian countries is significant, and predicted to increase [39]. This points to a need for public awareness that should be proactively addressed. Our data suggest pharmacists associated clients’ lack of awareness of this condition with inadequate public health campaigns; this is surprising when raising public awareness is indeed a role for pharmacists. This may be achieved using posters and health promotion initiatives in-store, and public health messages via the media.

Notwithstanding this, our data are unable to determine whether clients’ apparent lack of awareness about osteoporosis is a true lack of awareness, or lack of awareness of pharmacists’ contribution in this area. Clients may be aware of osteoporosis, but direct their enquiries to another health professional or search online for self-management options. In either case, pharmacist-led health promotion should be effective in both raising the profile of pharmacists’ role in osteoporosis management and public awareness. Our participating pharmacists appeared amendable to extending their services in this manner if more osteoporosis training were provided for them to develop competency. However, further research to explore the relevant stakeholders (such as doctors, patients and policy makers) regarding pharmacy-led osteoporosis disease state management services is required.

Previous studies indicated osteoporosis screening in community pharmacies has been well accepted by the public and other health care partners [15, 17, 40]. However, the accuracy of current screening tools (heel ultrasound and risk-assessment questionnaires) lacks verification, at least according to our participants, and would benefit from validation for their typical clients. Available research suggests heel ultrasound screening or a risk stratification algorithm (such as the fracture risk assessment tool [FRAX®]) must be based on device-specific cut-offs that are validated in the populations for which they are intended to be used [41]. The FRAX tool can be used to predict the probability to sustain the fractures within the next 10 years without the need to undergo a bone mineral density test. It can be downloaded from the Internet and is easy to use [42]. However, FRAX has not been validated in Malaysia. It has only been validated among the Malay, Chinese and Indian cohorts in Singapore. In the absence of a validated FRAX tool in Malaysia, use of the Singaporean FRAX tool is considered acceptable in Malaysia, due to similarities in their populations [43]. In terms of risk-screening questionnaires, the Osteoporosis Self-Assessment Tool for Asians is a simple and cost-effective tool validated for Asian populations [44, 45], and is supported by evidence from a trial in Thailand [22]. Regardless of evidence around the accuracy of diagnostic tools, our participants recognized the need to integrate screening measures and risk assessment into their service protocol.

Similar to published studies [46–48], the adoption of the disease state management services has been challenged by lack of knowledge, time and incentives, and limited inter-professional collaboration. Identifying barriers to pharmacists’ role transformation should be of prime concern, to find solutions and inform the expansion of these services. Pharmacists themselves can be barriers to their expanding roles and implementation of the disease state management services [49]. Our study confirms published research reporting lack of knowledge and time as intrinsic barriers to providing these services, along with staff, equipment, and information technology resources [50, 51]. Because of societal and normative expectations inherent in professional duty, pharmacists may have been compelled to respond to professional duty items in a socially desirable manner [52]. Pharmacists perceive osteoporosis is not their main concern compared to other conditions, such as diabetes and hypertension, since there is less demand from customers and lack of public campaign from government. Interventions such as targeted educational programs on osteoporosis using brochures or pamphlets may help pharmacists to disseminate osteoporosis information to clients [53].

A review of literature on the specific models for inter-professional collaboration between doctors and pharmacists found that trust and interdependency between doctors and pharmacists were critical determinants to the collaboration process [54, 55]. In the provision of osteoporosis disease state management, the pharmacist is unable to definitively diagnose osteoporosis, as this requires detailed clinical measures and judgement. Therefore, the pharmacist’s preliminary assessment requires diagnostic confirmation by the doctor. The pharmacist can then assist the doctor to counsel patients on appropriate use of their osteoporosis medications. In addition, pharmacists can provide advice regarding non-pharmacological improvement of bone health, such as lifestyle and diet modification [15]. However, due to the lack of dispensing separation in Malaysia, the roles of the doctors and pharmacists overlap, as both parties compete to dispense medications to patients [23]. Hence, the state of inter-professional collaboration between doctors and pharmacists in Malaysia is minimal. Further studies are required to ensure successful implementation of inter-professional osteoporosis disease state management in Malaysia. Other factors, such as education of policy makers, establishment of a reimbursement system and finding ways to encourage collaboration, should be addressed to achieve successful implementation.

### Strengths and limitations

The strength of this study is the application of qualitative methodology to collect rich, in-depth information about experiences and perceptions of community pharmacists on the provision of osteoporosis disease state management services. While the data, as presented, do not identify the source as individual interview or focus group, combining these methods was perceived to add balance to the data. The focus groups included study participants from heterogeneous settings, both independent and chain community pharmacies; although there are advantages to homogeneous groups [29], there are also benefits to carefully-managed heterogeneous groups and the richness of data that can be raised through interactions in the group [56]. Mixed groups of pharmacy owners and employees can foster idea stimulation through inclusion of more diverse participants who possess varying experiences and perspectives. There were no perceptible differences in responses between individual interview and focus group. However, in individual interviews, the facilitator had to probe the participant more, whereas discussion flowed more freely in the focus group discussions.

Findings from this sample may not be transferable to all community pharmacists in other localities, as there may be fewer opportunities for continuing education and patient referral. Conversely, inter-professional collaboration may be stronger in rural areas. Because our participants self-identified for the study, our data may reflect the views of those who feel more strongly on the issues. Furthermore, our recruitment strategy attracted involvement by pharmacists with and without experience in osteoporosis-related services. This provided a broad, rather than deep perspective to the issues. Another limitation of our study was the small number of pharmacists interviewed. In addition, there was no structured survey to quantify the opinions and practices of Malaysian community pharmacists regarding the provision of osteoporosis disease state management. With the establishment of a model service in osteoporosis management, future research is recommended to focus on reflections of the service providers.

Business-related financial issues were beyond the scope of this study, and this is suggested for later research in developing osteoporosis services or proposing a structure for remuneration. Another possible limitation is that participants may have given professionally-desirable responses. To minimize this, the interviewers highlighted their background and asked the respondents to be open-minded to share their experiences and opinions.

## Conclusions

This study found that the involvement of Malaysian community pharmacists in the provision of osteoporosis disease state management was minimal due to lack of public awareness and demand. Limited components of osteoporosis management were practiced. Pharmacist mainly counseled patients on intake of calcium and vitamin D, diet and lifestyle modification. Ad hoc osteoporosis screening using heel ultrasound was seldom conducted due to the unavailability of a suitable screening tool. Most identified FRAX as a suitable screening tool. Development and trial of collaborative osteoporosis disease state management services in community pharmacy could be facilitated by training, support and remuneration.

## Appendix 1

**Table 2:** The consolidated criteria for reporting qualitative studies (COREQ)

	Guide questions/description	Remarks	Page no.
Domain 1: Research team and reflexivity			
*a*). *Personal Characteristics*			
1. Interviewer/ facilitator	Which author/s conducted the interview or focus group?	Three authors (PSML, CJN and JN) conducted the in-depth interviews, while the focus group discussions were conducted by PSML and CJN.	3
2. Credentials	What were the researcher’s credentials? *E.g*. PhD, MD	The researchers’ credentials are as follows: JN: B Pharm PSML: PhD CJN: PhD LE: PhD	12
3. Occupation	What was their occupation at the time of the study?	The researchers’ occupations are as follows: JN: a postgraduate student/pharmacist PSML: academic / pharmacist CJN: academic / family physician LE: academic with Director of Research responsibilities.	12
4. Gender	Was the researcher male or female?	The researchers’ gender are as follows: JN: female PSML: female CJN: male LE: female	12
5. Experience and training	What experience or training did the researcher have?	JN attended a workshop on “how to conduct qualitative research” and “how to use NViVo software to analyse the data”. PSLM, CJN and LE are experienced researchers in qualitative studies and have collectively published numerous qualitative research articles.	12
*b*). *Relationship with participants*			
6. Relationship established	Was a relationship established prior to study commencement?	Only for the purposes of this research.	n/a
7. Participant knowledge of the interviewer	What did the participants know about the researcher? *e.g*. personal goals, reasons for doing the research	Some of the participants knew JN personally. The remaining participants did not know any of the researchers. However, all participants knew that the interview was for research purposes.	n/a
8. Interviewer characteristics	What characteristics were reported about the interviewer/facilitator? *e.g*. Bias, assumptions, reasons and interests in the research topic	The characteristics of each author have been reported in the section titled ‘Authors’ Information’.	12
Domain 2: study design			
*a*). *Theoretical framework*			
9. Methodological orientation and Theory	What methodological orientation was stated to underpin the study? *e.g*. grounded theory, discourse analysis etc	Interpretative descriptive and thematic analysis were applied.	2
*b*). *Participant selection*			
11. Method of approach	How were participants approached? *e.g*. face-to-face, telephone, mail, email	An invitation was posted on online social media (LinkedIn and Facebook). For those who expressed interest to participate, an email was then sent to confirm their interest, and to request their telephone number. The participant information sheet explaining the purpose of the study was then emailed to these potential participants.	3
12. Sample size	How many participants were in the study?	19 pharmacists were recruited.	4
13. Non-participation	How many people refused to participate or dropped out? Reasons?	Out of the 29 pharmacists approached, only 19 pharmacists completed the interviews (IDI = 9, FGD = 10). The reason for not participating were that they were busy.	4
*c*). *Setting*			
14. Setting of data collection	Where was the data collected? *e.g*. home, clinic, workplace	Data were collected at the pharmacists’ home or their workplace. Focus group discussions were conducted in a private meeting room located within a condominium residential area.	3
15. Presence of non-participants	Was anyone else present besides the participants and researchers?	No-one else was present besides the participants and the researchers.	n/a
16. Description of sample	What are the important characteristics of the sample? *e.g*. demographic data, date	The important characteristics of the samples were their age, gender, education background, number of years as a community pharmacist and whether they were working in independent or chain pharmacies.	3
*d*). *Data collection*			
17. Interview guide	Were questions, prompts, guides provided by the authors? Was it pilot tested?	A topic guide was prepared (Table 1), and pilot tested with 3 participants. Data from interviews conducted in the pilot test were included in the final analysis.	3
18. Repeat interviews	Were repeat interviews carried out? If yes, how many?	No repeat interviews were carried out.	3
19. Audio/visual recording	Did the research use audio or visual recording to collect the data?	Interviews were audio recorded.	3
20. Field notes	Were field notes made during and/or after the interview or focus group?	Field notes were made by JN after every interview and focus group discussion. These field notes were used to assist in the analysis of the transcribed audio recordings.	3
21. Duration	What was the duration of the interviews or focus group?	The duration of the in-depth interviews ranged from 30 to 60 min, while the focus group discussions were approximately 60 min.	3
22. Data saturation	Was data saturation discussed?	Data saturation was discussed in the methodology section.	3
23. Transcripts returned	Were transcripts returned to participants for comment and/or correction?	The transcripts were not returned to participants for comment or correction.	3
Domain 3: analysis and findings			
*a*). *Data analysis*			
24. Number of data coders	How many data coders coded the data?	Four authors worked in pairs (JN with CJN, and PSML with LE).	3
25. Description of the coding tree	Did authors provide a description of the coding tree?	The two pairs of authors coded two interviews line-by-line to develop an initial list of nodes, and to develop a framework. This framework was then used to code the next transcript. Coding discrepancies were resolved by discussion between representatives of the pairs until consensus was reached. The lists of nodes were used as the final coding framework for the remaining transcripts. New nodes emerging during coding were added to the list upon consultation with the research team. The lists of nodes were regrouped into larger categories as themes emerged from the data. This systematic approach to the analysis established an audit trail from the transcripts of raw data through to the final interpretation. Analysis was undertaken concurrently with data collection to check for data saturation.	3
26. Derivation of themes	Were themes identified in advance or derived from the data	The themes were derived from the data during analysis.	4
27. Software	What software, if applicable, was used to manage the data?	NVivo 10 was used to manage the data.	3
28. Participant checking	Did participants provide feedback on the findings?	No, participants did not provide any feedback on the findings.	n/a
*b*). *Reporting*			
29. Quotations presented	Were participant quotations presented to illustrate the themes / findings? Was each quotation identified? *e.g*. participant number	Yes, quotations were presented and identified by the respondent’s age and gender.	4-7
30. Data and findings consistent	Was there consistency between the data presented and the findings?	Yes, there was consistency between the data presented and the findings.	4-7
31. Clarity of major themes	Were major themes clearly presented in the findings?	Yes, major themes was clearly presented in the findings.	4-7
32. Clarity of minor themes	Is there a description of diverse cases or discussion of minor themes?	No, there was no descriptions of the diverse cases or discussion of minor themes presented.	n/a

## Appendix 2

**Table 3:** Quantification of nodes

Categories	Themes	Frequency of nodes
Provision of osteoporosis disease state management services by community pharmacists	Counselling regarding calcium	13
	Counselling on the risk factors for osteoporosis	13
	Counselling on how to take their osteoporosis medication	10
	Counselling on lifestyle changes	8
	Use of heel ultrasound as a means of screening for osteoporosis	6
	Referral of patients who are at high risk of osteoporosis to a doctor	6
	Use of accurate and validated screening tools to screen for osteoporosis	1
Barriers to the provision of osteoporosis disease state management services	Lack of public awareness	17
	Lack of customer demand	16
	Lack of accurate and validated screening tools for osteoporosis	7
	Lack of pharmacists’ knowledge	7
	Lack of pharmacists’ time to counsel on bone health	6
	Lack of collaboration between doctors and pharmacists	5
	Lack of continuity of care	3
Facilitators in the provision of osteoporosis disease state management services	Public health campaigns on osteoporosis	17
	Continuing pharmacists’ education support on osteoporosis	13
	Inter-professional collaboration	12
	Support from the pharmaceutical industry	10
	A simple and unbiased education material on osteoporosis for customers	7
	Reimbursement from the government to pharmacists for the additional service provided on osteoporosis disease state management services	4

## Abbreviations

DXA: Dual-energy x-ray absorptiometry
FRAX®: Fracture risk assessment tool
IBM: Integrated behavior model
